# Stability and anti-tumor effect of oncolytic herpes simplex virus type 2

**DOI:** 10.18632/oncotarget.25122

**Published:** 2018-05-15

**Authors:** Yang Wang, Jing Jin, Zhen Wu, Sheng Hu, Han Hu, Zhifeng Ning, Yanfei Li, Yuting Dong, Jianwen Zou, Zeyong Mao, Xiaotai Shi, Huajun Zheng, Shuang Dong, Fuxing Liu, Zhizheng Fang, Jiliang Wu, Binlei Liu

**Affiliations:** ^1^ National “111” Center for Cellular Regulation and Molecular Pharmaceutics, Key Laboratory of Fermentation Engineering (Ministry of Education), Hubei Provincial Cooperative Innovation Center of Industrial Fermentation, Hubei Key Laboratory of Industrial Microbiology, Hubei University of Technology, Wuhan, 430068, Hubei, China; ^2^ Department of Medical Oncology, Hubei Cancer Hospital, Wuhan, 430079, Hubei, China; ^3^ Basic Medicine College, Hubei University of Science and Technology, Xianning, 437100, Hubei, China; ^4^ College of Pharmacology, Hubei University of Science and Technology, Xianning, 437100, Hubei, China; ^5^ Wuhan Binhui Biotechnology Co., Ltd., Wuhan, 430075, Hubei, China; ^6^ Shanghai-MOST Key Laboratory of Health and Disease Genomics, Chinese National Human Genome Center at Shanghai, 201203, Shanghai, China; ^7^ Hubei Provincial Key Laboratory of Cardiocerebrovascular and Metabolic Diseases, Hubei University of Science and Technology, Xianning, 437100, Hubei, China

**Keywords:** herpes simplex virus type 2, stability, anti-tumor effect

## Abstract

Oncolytic virotherapy is a new therapeutic strategy based on the inherent cytotoxicity of viruses and their ability to replicate and spread in tumors in a selective manner. We constructed a new type of oncolytic herpes simplex virus type 2 (oHSV-2, named OH2) to treat human cancers, but a systematic evaluation of the stability and oncolytic ability of this virus is lacking. In this study, we evaluated its physical stability, gene modification stability and biological characteristics stability, including its anti-tumor activity in an animal model. The physical characteristics as well as genetic deletions and insertions in OH2 were stable, and the anti-tumor activity remained stable even after passage of the virus for more than 20 generations. In conclusion, OH2 is a virus that has stable structural and biological traits. Furthermore, OH2 is a potent oncolytic agent against tumor cells.

## INTRODUCTION

Cancer is a type of malignant disease that threatens human health [1]. Although medical science and technology have advanced quickly and the molecular mechanisms of carcinogenesis are being increasingly revealed, the total five-year survival rate of human cancers remains at a low level [2]. Surgical resection of primary tumors combined with chemotherapeutics and different types of radiotherapy remain the mainstream treatments [3]. In view of the shortcomings of the existing therapies, researchers have sought to exploit new therapeutic modes to conquer this deadly disease. Herpes simplex virus (HSV) is an enveloped, double-stranded DNA virus that causes cold sores in its wild-type form [4]. HSV is a naturally neurotropic virus that is spread by contact with the skin, efficiently taken up by nerve terminals and transported along axons to establish a persistent (latent) state in sensory neurons of the dorsal root ganglion (DRG) or trigeminal ganglion. Herpes viruses are extremely successful pathogens that have coevolved with their mammalian hosts over the past 60–80 million years [5]. There are eight human herpes viruses that can be subdivided into three subfamilies: (i) Alphaherpesvirinae; (ii) Betaherpesvirinae; and (iii) Gammaherpesvirinae [6]. Herpes virus infection is associated with a large array of clinical manifestations, spanning from skin/mucosal lesions to several malignancies. Herpes simplex virus types 1 and 2 (HSV-1 and HSV-2) belong to the Alphaherpesvirinae subfamily [7]; human cytomegalovirus (HCMV) belongs to the Betaherpesvirinae subfamily [8]; and Epstein–Barr virus (EBV) and human herpes virus 8 [HHV-8, also known as Kaposi’s sarcoma-associated herpes virus (KSHV)] both belong to the Gammaherpesvirinae subfamily [9, 10]. Alphaherpesviruses, especially the human HSV-1 and HSV-2 serotypes, infect humans via contact of the oral or genital mucosa with viral particles. From the mucosa, the virus migrates and establishes a latent infection in sensory or autonomic neurons near the primary site of infection [7, 11]. HSV-1 was one of the first viruses to be developed into a recombinant oncolytic virotherapeutic vector. One major reason for therapy failure is an inability to target tumor cells specifically, possibly resulting in harm to normal, non-transformed, surrounding cells. Although viruses may be harmful to human health, after transformation to modify their molecular structures, such as deletion of morbigenous factors, all types of viruses have been used to diagnose and treat human cancers. The first reports of oncolytic viral therapy date back to the 1950s and used the intriguingly named Bunyamwera, West Nile, and Semliki Forest viruses [12]. Currently, many types of oncolytic viruses, including vaccinia virus, adenovirus, herpes simplex virus, reovirus, and Newcastle disease virus, have been constructed using genetic engineering methods and have entered the preclinical and clinical research phases [13–16]. Modified HSV (herpes simplex virus) is one virus type that is utilized to diagnose and treat cancers. To date, several HSV mutants, including Oncovex^GM-CSF^ (T-vec) [17], 1716 [18, 19], G207 [20, 21], NV1020 [22, 23], HF10 and G47Δ, have either completed or entered phase I, II, and III clinical trials to treat cancers of different degrees of malignancy from different tissue origins, such as melanoma, breast cancer and glioma. Because of its replication selectivity, oncolytic HSV was initially localized to treat superficial metastatic tumors, such as metastatic melanoma, as mentioned above, although determining the proper treatment scope requires further investigation. The stability of genetically engineered viruses is another important biological problem. However, to the best of our knowledge, few data are available on this topic. Stability is directly linked to the biological characteristics of the virus, treatment efficiency, storage methods and validity period [24]. In our previous study, we constructed a novel oncolytic herpes simplex virus type 2 (HG52/ICP34.5-/ICP47-) that expressed human GM-CSF (OH2). In this study, we evaluated the stability of the physical characteristics, gene modifications, and biological characteristics, including its anti-tumor activity, in an animal model. Our results indicate that the physical and gene modification characteristics of OH2 were stable and that its oncolytic activity remained unchanged even after passage of the virus for more than 20 generations. The results demonstrate that OH2 possesses stable structural and biological traits and is a potent oncolytic agent for tumor virotherapy.

## RESULTS

### Growth curves, TEM structures and gene modification stability of the 4^th^, 10^th^, and 20^th^ OH2 virus generations are similar

**Figure 1:**
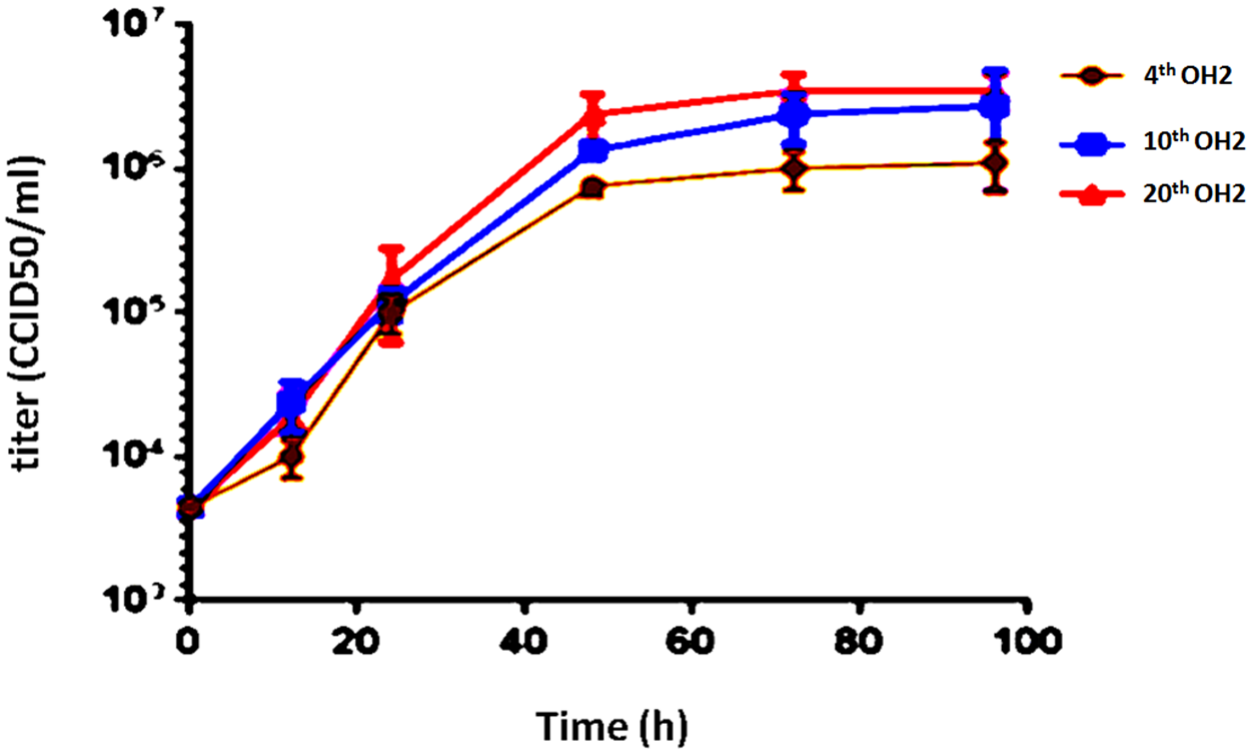
Growth curves of the 4^th^, 10^th^, and 20^th^ OH2 generations

**Figure 2:**
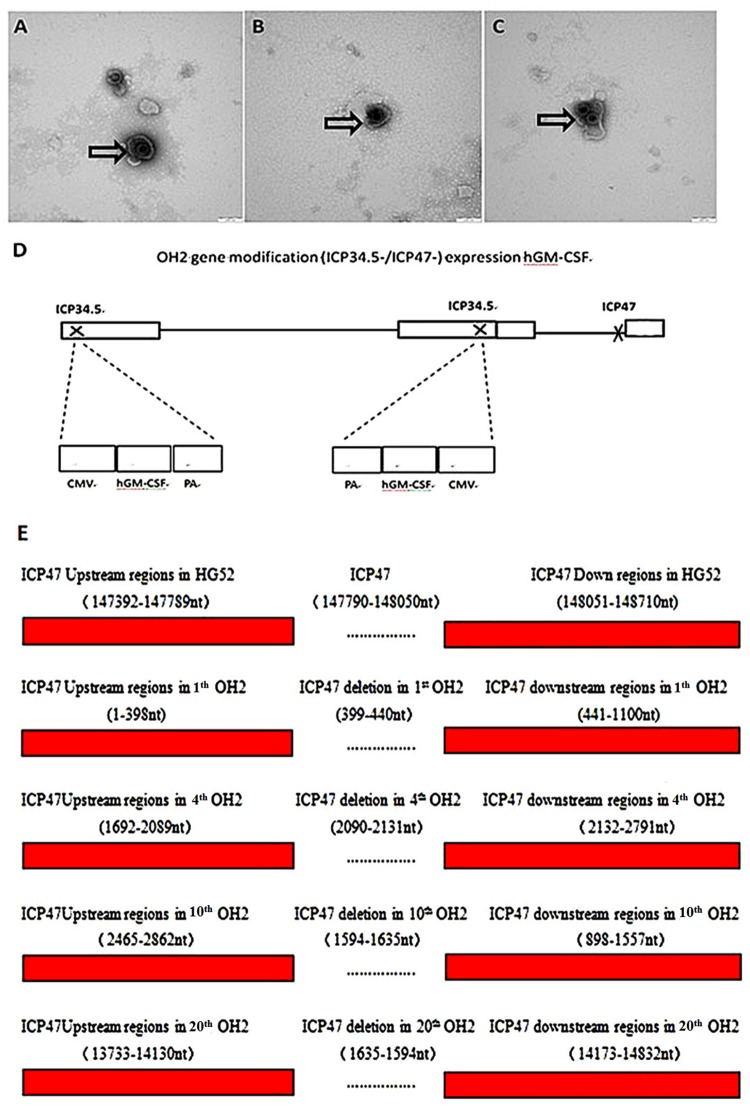
**(A, B, C)** TEM micrographs of the 4^th^ (A), 10^th^ (B) and 20^th^ (C) OH2 generations. The viral particles were observed under a transmission electron microscope after negative staining with phosphotungstic acid. **(D)** OH2 gene modification (ICP34.5-/ICP47-) for hGM-CSF expression. **(E)** Flanking sequence of ICP47 in 1^th^, 4^th^, 10^th^ and 20^th^ OH2.

The growth curves of the different OH2 generations were very similar, showed an upward trend in the 0-48 h period and remained stable from 48-72 h. No significant differences in the titers of the 4^th^, 10^th^, and 20^th^ generations were found at the same time points (Figure 1). The viral particles were observed under a transmission electron microscope after negative staining with phosphotungstic acid. Each viral particle was spherical and approximately 100-120 nm in diameter (Figure 2A, 2B, 2C). OH2 is a novel oncolytic herpes simplex virus type 2 (HG52/ICP34.5-/ICP47-) that expresses human GM-CSF. The gene modifications introduced into OH2 are shown in Figure 2D. Sequence analysis revealed that no single mutations occurred in the hGM-CSF gene in the 4^th^, 10^th^ and 20^th^ generation OH2 viruses. Reverse mutations in the ICP34.5 and ICP47 genes did not occur after OH2 was passaged in Vero cells for 20 generations (Figure 2E, Table 1).

**Table 1:** Flanking sequence of ICP34.5 and the insertion position of hGM-CSF in the 1^th^, 4^th^, 10^th^ and 20^th^ OH2 virus generations

Position of base sequence	1^th^ OH2	4^th^ OH2	10^th^ OH2	20^th^ OH2
**Scaffold**	6	8	8	4
**34.5A (nt)**	3715-5012	1663-2969	612-1917	2026-3332
**34.5B (nt)**	6505-7089	1-179	3402-3598	1-542
**hGM-CSF (nt)**	5753-6187	497-931	2650-3084	860-1294
**CMV (nt)**	5062-5716	968-1622	1959-2613	1331-1985
**BGH-Poly A (nt)**	6228-6459	225-456	3125-3356	588-819

### Stability and activity of virus-expressed hGM-CSF

**Figure 3:**
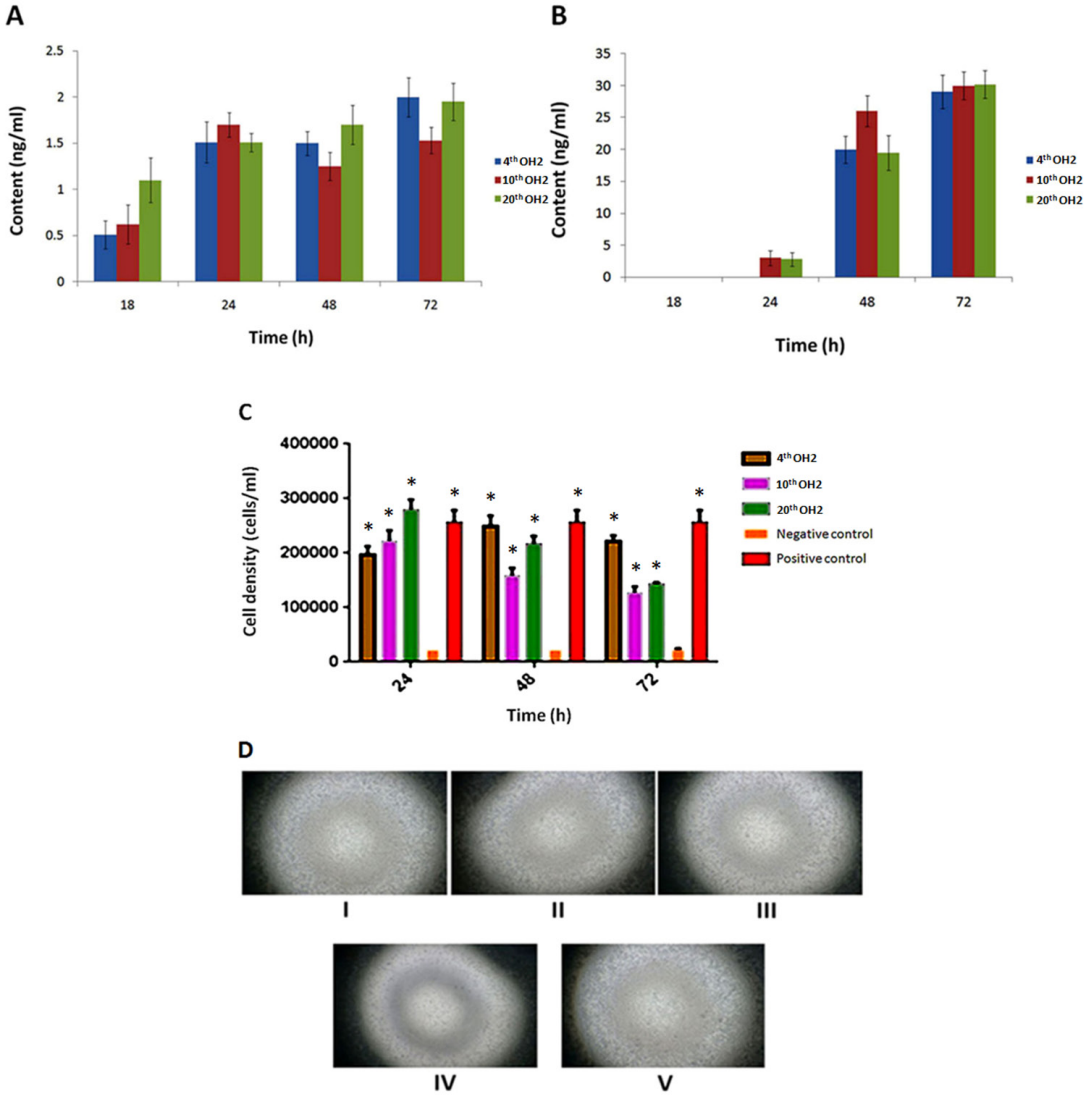
**(A)** hGM-CSF content in A549 cells infected with 4^th^, 10^th^, and 20^th^ generation OH2 viruses. **(B)** hGM-CSF content in Vero cells infected with 4^th^, 10^th^, and 20^th^ generation OH2 viruses. **(C)** Activity of virus-expressed hGM-CSF. ^*^ *p*<0.05 compared with the negative control. **(D)** Images of TF-1 proliferation stimulated by hGM-CSF expressed from different OH2 generations. I: The growth state of TF-1 cells stimulated by the viral supernatant produced by 4^th^ generation OH2. II: The growth state of TF-1 cells stimulated by the viral supernatant produced by 10^th^ generation OH2. III: The growth state of TF-1 cells stimulated by the viral supernatant produced by 20^th^ generation OH2. IV: The growth state of TF-1 cells in culture medium without GM-CSF (negative control group). V: The growth state of TF-1 cells in culture medium containing GM-CSF (positive control group).

The hGM-CSF content was detected using the sandwich ELISA method in A549 and Vero cells infected with viruses from different generations. No significant differences were found between viruses from different generations (Figure 3A, 3B). We used TF-1 cells to evaluate the activity of virus-expressed hGM-CSF. The cell densities at 72 h were 2.5×10^4^/mL in the negative control group, 2.6×10^5^/mL in the positive control group (10 ng/mL of clinical grade hGM-CSF), and greater than 1.2×10^5^/mL for all of the tested supernatant groups (hGM-CSF produced by the 4^th^, 10^th^, and 20^th^ OH2 generations). Statistical analysis of the cell densities indicated a significant difference between the negative control group and tested supernatant groups or positive control group (Figure 3C, 3D).

### Oncolytic ability of OH2 *in vitro*

**Figure 4:**
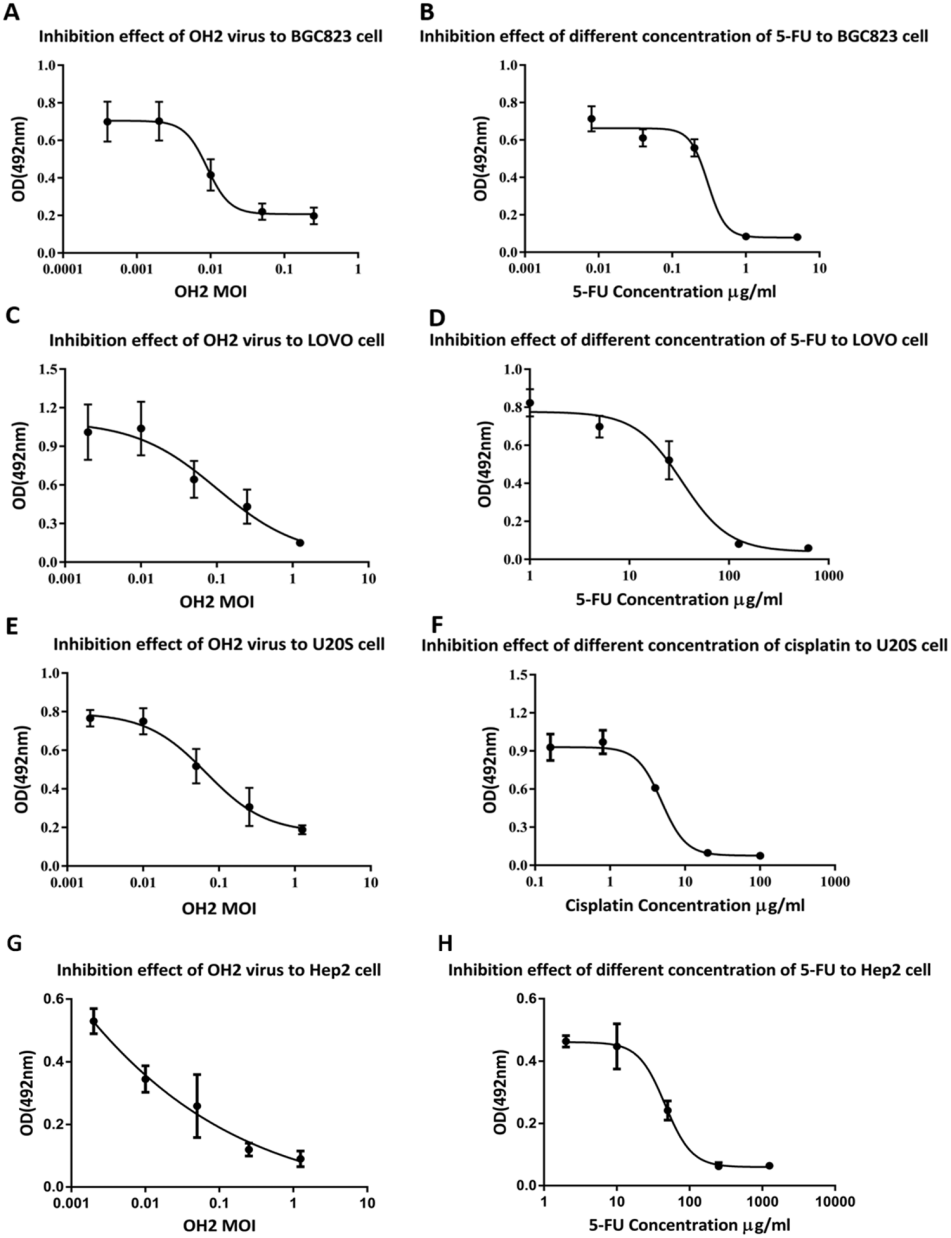
Oncolytic ability of OH2 *in vitro* **(A)** Inhibitory effect of OH2 on BGC823 cells *in vitro*. **(B)** Inhibitory effect of different concentrations of 5-Fu on BGC823 cells *in vitro*. **(C)** Inhibitory effect of OH2 on LoVo cells *in vitro*. **(D)** Inhibitory effect of different concentrations of 5-Fu on LoVo cells *in vitro*. **(E)** Inhibitory effect of OH2 on U20S cells *in vitro*. **(F)** Inhibitory effect of different concentrations of cisplatin on U20S cells *in vitro*. **(G)** Inhibitory effect of OH2 on Hep2 cells *in vitro*. **(H)** Inhibitory effect of different concentrations of 5-Fu on Hep2 cells *in vitro*.

The MTT method was used to analyze the oncolytic ability of OH2 in BGC823, LoVo and U20S cells, which are expressed as MOI_IC50_ values (calculated using SPSS). The OH2 virus MOI_IC50_ values were 0.021±0.001 (BGC823), 0.151±0.017 (LoVo), 0.245±0.103 (U20S) and 0.039±0.024 (Hep2); whereas the chemotherapy drug IC_50_ values were 0.290±0.057 μg/mL (BGC823), 27.343±0.791 (LoVo), 6.617±0.721 (U20S) and 16.5±0.75 (Hep2). The MOI_IC50_ values of the OH2 virus in the three tumor cell lines were very low, indicating that OH2 had a good killing effect on these tumor cells (Figure 4).

### Oncolytic ability of OH2 *in vivo*

**Figure 5:**
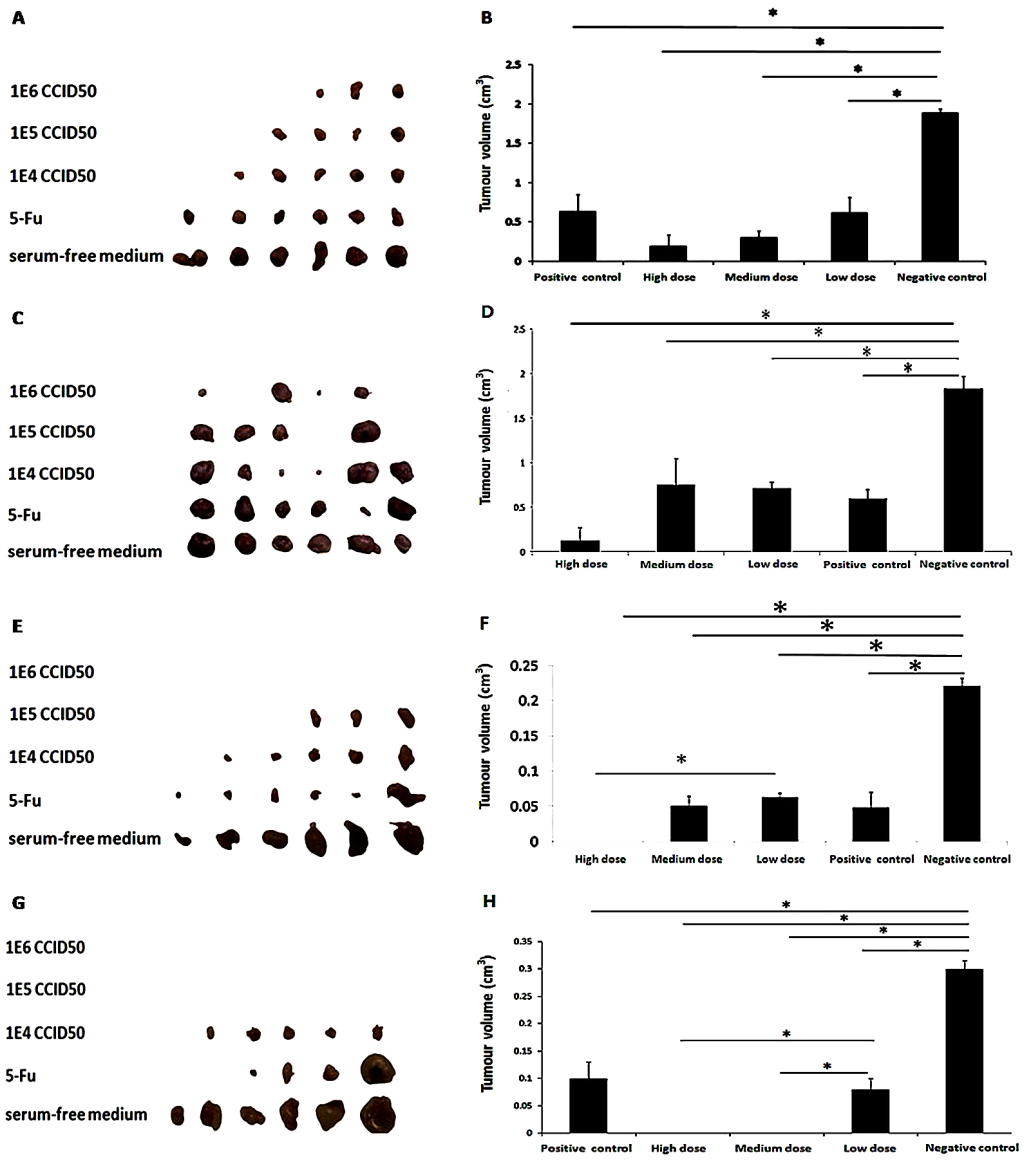
Oncolytic ability of OH2 *in vivo* **(A, B)** Inhibitory effect of OH2 on BGC823 cells *in vivo* (n=6). **(C, D)** Inhibitory effect of OH2 on CT26 cells *in vivo* (n=6). **(E, F)** Inhibitory effect of OH2 on LoVo cells *in vivo* (n=6). **(G, H)** Inhibitory effect of OH2 on Hep2 cells *in vivo* (n=6).

In the BGC823 group, the results (Figure 5A and 5B) showed that the relative tumor growth rates (T/C) of OH2 in the high-, medium- and low-dose groups were 10.15%, 15.17%, and 29.43%, respectively. A significant difference in the tumor volume was found between the OH2 group and negative control group (*p*<0.05). The curative effect was strongest for the high-dose (1E6) group, but the low-dose group also had a good anti-tumor effect. In the CT26 group, the relative tumor growth rates (T/C) of OH2 in the high-, medium- and low-dose OH2 groups and positive control group were 7%, 38.64%, 37.77%, and 33.8%, respectively. A significant difference in tumor volume was found between the OH2 group and negative control group (*p*<0.05). The curative effect was strongest for the high-dose (1E6) group, but the low-dose group also had a good anti-tumor effect (Figure 5C and 5D). In the LoVo group, the relative tumor growth rates (T/C) of OH2 in the high-, medium- and low-dose groups and in the positive control group were 0%, 25.18%, 28.27%, and 25.24%, respectively. A significant difference (*p*<0.05) in the average tumor volume was found between the OH2 group and negative control group (*p*<0.05). The curative effect was strongest for the high-dose (1E6) group, but the low-dose group also had a good anti-tumor effect (Figure 5E and 5F). In the Hep2 group, the relative tumor growth rates (T/C) of OH2 in the high-, medium- and low-dose groups and positive control group were 0%, 0%, 30.92%, and 33.45%, respectively. A significant difference (*p*<0.05) in the average tumor volume was found between the OH2 group and negative control group. A significant difference (*p*<0.05) in average tumor volume was found between high-dose OH2 group and Low-dose OH2 group (Figure 5G and 5H).

### Inhibitory effects of the 4^th^, 10^th^ and 20^th^ OH2 virus generations on cancer

**Figure 6:**
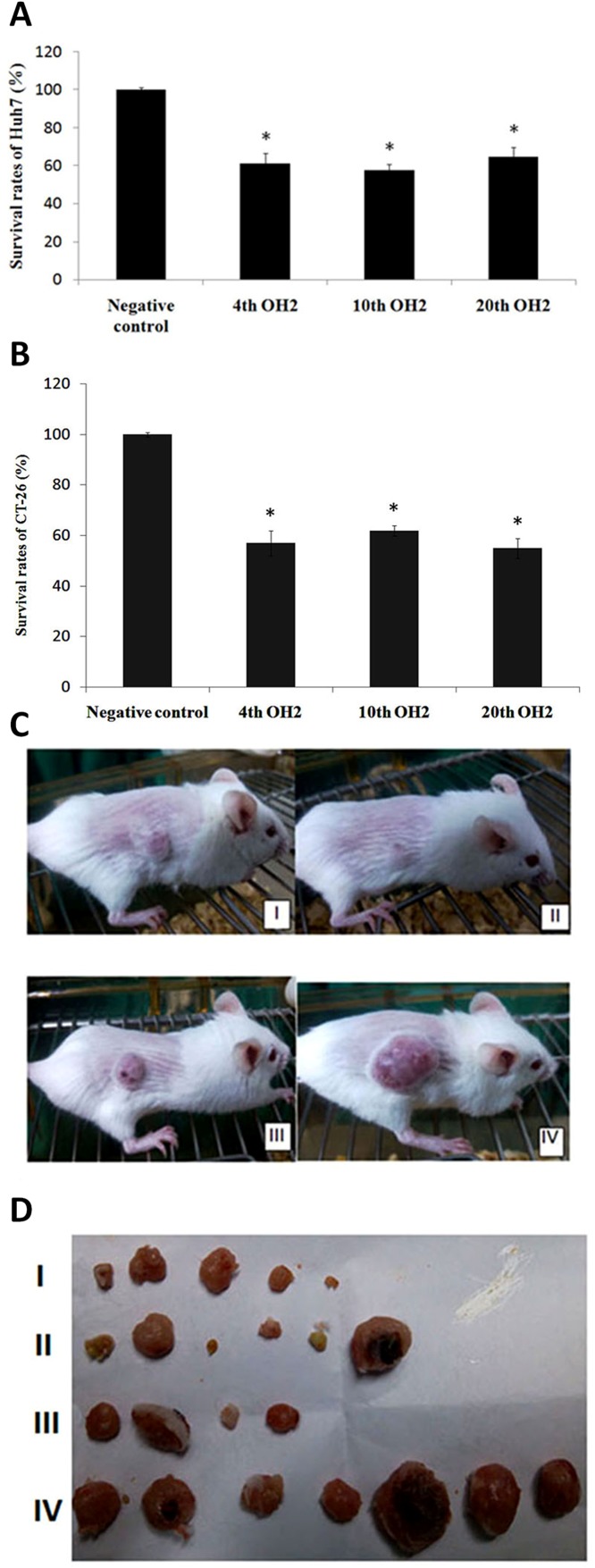
The inhibitory effects of the 4^th^, 10^th^, and 20^th^ OH2 virus generations on cancer **(A)** Survival rates of Huh7 cells. ^*^ *p*<0.05 compared with the negative control, **(B)** Survival rates of CT-26 cells. ^*^ *p*<0.05 compared with the negative control, **(C)** Representative CT-26 tumor-bearing mice from the 4^th^ (I), 10^th^ (II), and 20^th^ (III) generation OH2-treated groups and control group (IV). **(D)** CT-26 tumors from the 4^th^ (I, n=7), 10^th^ (II, n=7), and 20^th^ (III, n=7) generation OH2-treated groups and control group (IV) at the end of the experiment.

Compared to the control group (untreated group), the 4^th^, 10^th^ and 20^th^ generation OH2 viruses all had significant inhibitory effects on Huh7 human hepatoma cells or mouse colon cancer cells (CT-26) (Figure 6A and 6B). At the end of treatment in the CT-26 model, the average tumor volumes of mice treated with the 4^th^, 10^th^, and 20^th^ OH2 generations and control group were 93.2 mm^3^ (I: tumor inhibitory rate (TIR) of 90.4%), 255.1 mm^3^ (II: TIR of 74.1%), 213.4 mm^3^ (III: TIR of 79.9%), and 935.6 mm^3^ (IV), respectively. Moreover, no deaths occurred in mice (Figure 6C and 6D).

## DISCUSSION

Oncolytic viruses are emerging as important agents in cancer treatment. A diverse range of viruses has been investigated as potential cancer therapeutics [25]. Oncolytic HSV selectively replicates within tumors and directly destroys tumor cells by virus-induced cell lysis, a mechanism that is obviously different from routine cancer therapies, such as chemotherapy and radiotherapy [26, 27]. T-Vec (talimogene laherparepvec), a second-generation oncolytic herpes simplex virus type 1 (HSV-1) armed with GM-CSF, with deletions in the ICP34.5 and ICP47 genes, was recently approved as the first oncolytic virus drug in the USA and Europe [28]. The phase III trial of T-Vec demonstrated that local intralesional injections of T-Vec in advanced malignant melanoma patients not only suppressed the growth of tumors but also acted systemically to prolong overall survival [29].

HSV-1 and HSV-2 are highly prevalent viruses that cause a variety of diseases, from cold sores to encephalitis. Both viruses establish latency in peripheral neurons, but the molecular mechanisms facilitating the infection of neurons are not fully understood. HSV-1 is normally transmitted during childhood and is linked to facial herpes, whereas HSV-2 is normally sexually transmitted and associated with genital herpes. HSV-2 is considered to be more potent than HSV-1 in the destruction of tumors [30]. In our previous study, we constructed a novel oncolytic herpes simplex virus OH2 (with deletions in ICP34.5 and ICP47 and expression of human GM-CSF) [31]. The ICP34.5 gene in HSV is mainly responsible for cancer-selective replication and attenuation of pathogenicity. Because the ICP34.5 gene functions negate the host cell’s shut-off of protein synthesis upon viral infection, inactivation of ICP34.5 renders the virus unable to replicate in normal cells. However, because cancer cells are defective in the shut-off response, ICP34.5-deficient HSV can still replicate in cancer cells [32]. The ICP47 gene functions to antagonize the host cell’s transporter associated with antigen presentation. Therefore, deletion of the gene precludes the down regulation of MHC class I expression, which should enhance antitumor immune responses. The deletion in the ICP47 gene also results in immediate early expression of the neighbor US11 gene, which results in enhanced viral replication in cancer cells [33].

In this study, we investigated the gene modification stability (with deletions in ICP34.5, ICP47 and insertion of a human GM-CSF expression cassette) of the 4^th^, 10^th^ and 20^th^ generations of OH2. No single mutation in the hGM-CSF gene was found in the 4^th^, 10^th^, and 20^th^ generations of OH2 viruses. Reverse mutations in the ICP34.5 and ICP47 genes did not occur after passage of OH2 in Vero cells for 20 generations. The nucleotide change mutation introduced safety issues [27]. For instance, ICP6, which encodes the large subunit of the ribonucleotide reductase, is essential for viral DNA replication. HSV with the ICP6 mutation can only replicate in rapidly dividing cancer cells but not in quiescent normal cells. If a reversion mutation, such as nucleotide substitution, occurs in the ICP6 gene, the genetically engineered HSV may replicate not only in cancer cells but also in normal cells and thus lose its ability to kill cancer cells selectively [34]. A true example of a mutation-driven property change occurred with a single base pair change in the Chikungunya virus (CHIKV) E1 gene, which caused CHIKV infectivity to significantly increase [35].

Continuous passages may influence the virulence, growth property and even viral structure of a virus. For example, mutation of the HSV-1 KOS sub-strains appeared after passaging. Five coding changes that could potentially explain the KOS1.1 phenotypic properties of increased replication at high temperature and decreased neuroinvasiveness were identified. The use of a high temperature and selection of a single plaque during the passage of the KOS stain could have resulted in the increase of the mutation frequency of the virus genome [36]. However, neither coding nor non-coding mutations occurred in the OH2 gene modification area for up to 20 passages. Furthermore, we showed that the growth curves and TEM structures of the 4^th^, 10^th^ and 20^th^ generation OH2 viruses were highly similar. Gene modification stability and a stable virus structure in an oncolytic virus during continuous passages are important issues that should be taken into account carefully before the virus is applied clinically.

In addition to the above stability analyses, the anti-tumor effect of OH2 is another important issue. The comparison of the anti-tumor effect of the 4^th^, 10^th^ and 20^th^ generation OH2 viruses indicated that OH2 has a good ability to kill tumor cells and that the 4^th^, 10^th^ and 20^th^ OH2 generations retained an equivalent anti-tumor effect.

Recent research analyzed the stability of an adenovirus-derived oncolytic agent and demonstrated that this radioactive iodine-labelled, prostate cancer-specific, recombinant oncolytic adenovirus can stably maintain its radioactivity under specific conditions. However, the general stability characteristics regarding the structural and biological properties of oncolytic viruses were not elucidated [37]. The gene modifications, TEM structure, inserted gene expression/function and anti-tumor effect of various OH2 generations were analyzed comparatively in our lab. Our study is the first to report the structural and biological stability of an OH2 virus even after passaging for 20 generations. We conclude that OH2 is a potent and stable oncolytic agent for cancer therapy.

## MATERIALS AND METHODS

### Ethics statement

All animal tissue collection procedures were performed according to protocols approved by the Hubei Province, PR China, Biological Studies Animal Care and Use Committee.

### Cells and virus

The cancer cell lines Vero, LoVo, Huh7, U20s, BGC823 and Hep2 were purchased from the American Type Culture Collection (ATCC, Virginia, USA). Cells were cultured in DMEM/F12 medium supplemented with 5% NBS, 100 μg/mL of streptomycin, and 100 U/mL of penicillin and incubated at 37°C with 5% CO_2_ and saturated humidity. The TF-1 human leukemia cell line was purchased from the Cell Resource Center of Peking Union Medical College and was cultured in RPMI 1640 medium supplemented with 10% FBS. The A549 human lung cancer cell line and CT26 mouse colon cancer cell line were purchased from the Cell Resource Center of Peking Union Medical College and were cultured in DMEM/F12 medium supplemented with 10% FBS. OH2 oncolytic herpes virus type 2 was constructed from the HG52 strain.

### Mice and reagents

Six-week-old female BALB/c and BALB/c nude mice and purchased from the Hubei Food and Drug Safety Evaluation Center and Beijing HuaFukang Bioscience co.inc were housed under specific pathogen-free conditions.

### Detection of virus growth

First, OH2 was passaged in Vero cells for 20 generations. Second, Vero cells were seeded onto 6-well plates, incubated overnight, counted and infected with the 4^th^, 10^th^, and 20^th^ generations of OH2 at an MOI=0.02. Third, the viruses were harvested at different time points, and the viral titers were determined using the CCID_50_ method. Finally, growth curves were drawn based on the viral titers that were calculated using the Karber formula.

### Detection of hGM-CSF expression by the OH2 virus

A549 and Vero cells in 6-well plates were counted and infected with the 4^th^, 10^th^ and 20^th^ generations of OH2 at an MOI=0.1. The supernatants were harvested at different time points for measurement of the hGM-CSF contents using the ELISA sandwich method. A mouse anti-hGM-CSF monoclonal antibody was diluted to 8 μg/mL with carbonate buffer (pH 9.6) and used to coat microtiter plates at 4°C. Twelve hours later, the operating wells coated with antibody were blocked with PBS buffer containing 1% BSA for 1 h and then washed with PBS buffer three times. Supernatants from Vero and A549 cells infected with OH2 (experimental group), the hGM-CSF positive standard diluted to different concentrations (125 pg/mL, 250 pg/mL, 500 pg/mL, 1 ng/mL, 2 ng/mL and 4 ng/mL, the control group) and the diluent (negative control group, 50 μl/well) were added to the plates. hGM-CSF (4 ng/mL) was added to the wells without antibody as a blank control (50 μl/well). All samples to be tested were incubated at 37°C for 1 h. All wells were washed and reacted with a biotin-labeled hGM-CSF monoclonal antibody (0.1 μg/mL) at 37°C (50 μl/well). One hour later, the wells were washed and streptavidin-HRP (0.1 μg/mL, 60 μl/well) was added. The plates were left to rest at room temperature for 30 minutes, washed again and then incubated with a TMB solution (50 μl/well) in the dark for 15 minutes. Sulfuric acid (2 mol/L, 50 μl/well) was added, and the light absorption value of each well was detected at a wavelength of 405 nm.

### Evaluation of hGM-CSF activity in different generations of OH2

In this study, the TF-1 cell culture method was used to detect the activity of hGM-CSF expressed by the 4^th^, 10^th^ and 20^th^ OH2 generations. TF-1 cells in 6-well plates were divided into three groups: TF-1 cell growth was stimulated by adding supernatants from Vero cells infected with viruses from the different generations (experimental group); hGM-CSF (the positive control group); and culture medium (the negative control group). The TF-1 cell numbers were counted, and cell growth was measured under a microscope at 24, 48 and 72 h after culturing.

### Transmission electron microscopy

The samples (4^th^, 10^th^ and 20^th^ OH2 generations) for transmission electron microscopy (TEM) were prepared by placing the samples into epoxy capsules and then curing the epoxy at 70°C for 24 h in vacuum. The cured epoxies were then sectioned into 9-mm-thick slices, and a layer of carbon that was approximately 3-nm thick was deposited onto each slice on a 200-mesh size copper net. TEM photographs were taken on a HITACHI-H7650 transmission electron microscope at an acceleration voltage of 120 kV.

### High-density pyrosequencing and genome sequence assembly

Genome sequencing for the 1^th^, 4^th^, 10^th^ and 20^th^ generations was conducted using the Roche GS FLX system. Assembly was performed using the GS *de novo* Assembler software (http://www.454.com/). The relationship of the contigs was determined by multiplex PCR. The Phred, Phrap, and Consed software packages (http://www.genome.washington.edu) were used for final assembly and editing.

### Oncolytic ability of OH2 *in vitro*

The BGC823, LoVo, U20S and Hep2 tumor cell lines were used in the MTT experiments. To evaluate the effect of the intensity of the OH2 virus and its sensitivity to the different types of tumor cells, human tumor cells were cultured with different concentrations of the OH2 virus, and the 50% OH2 viral inhibition MOI (MOI_IC50_) was determined via the MTT method (MOI, the multiplicity of infection, refers to the units of active virus needed to infect one cell) and compared with positive control drugs. The Huh7 cell line and CT-26 cell line were used to evaluate the oncolytic ability of the 4^th^, 10^th^ and 20^th^ OH2 generations. The survival rate of Huh7 cells and CT-26 cells after OH2 infection was determined via the MTT method.

### Oncolytic ability of OH2 *in vivo*

BGC823 cells at 1E6 were injected subcutaneously into the right flanks of female BALB/c nude mice to induce tumor growth. The animals were randomly divided into five groups; each group contained six mice. Three different doses of OH2 (1E6, 1E5 and 1E4 CCID_50_) were injected into the tumor. The positive and negative control groups received 5 mg/mL of 5-fluorouracil and serum-free medium, respectively. The injection volume was 100 μl, and mice received injections once every three days for 3 consecutive treatments. A CT26 mouse colon cancer model was successfully established. The animals were randomly divided into five groups; each group contained six mice, and three different doses of OH2 (1E6, 1E5 and 1E4 CCID_50_) were injected into the tumor. The control groups were treated with 5 mg/mL of 5-fluorouracil (positive control group) and serum-free medium (negative control group). The injection volume was 100 μl, and mice were injected once every three days for 3 consecutive treatments. LoVo cells were subcutaneously injected into the right flanks of female BALB/c nude mice to induce tumor formation. Tumor-bearing animals were randomly divided into groups. Subsequently, the tumors of the three OH2-treated groups were injected with three different doses of OH2 (1E6, 1E5 and 1E4 CCID_50_); the control groups were treated with 5 mg/mL of 5-fluorouracil (positive control group) and serum-free medium (negative control group). The injection volume was 100 μl, and administration was performed three times (at days 0, 3 and 6). Hep2 cells were subcutaneously injected into the right flanks of female BALB/c nude mice to induce tumor formation. The tumor-bearing animals were randomly divided into groups. Subsequently, the tumors in the three OH2-treated groups were injected with three different doses of OH2 (1E6, 1E5 and 1E4 CCID_50_), and the control groups were treated with 5 mg/mL of 5-fluorouracil (positive control group) and serum-free medium (negative control group). The injection volume was 100 μl, and administration was performed three times (at days 0, 3 and 6). CT-26 cells at 1E6 were injected subcutaneously into the right flanks of female BALB/c nude mice to induce tumor formation. The animals were randomly divided into groups. The 4^th^, 10^th^ and 20^th^ OH2 passages (1E6 CCID_50_) were injected into the tumors, with serum-free medium serving as a negative control. The injection volume was 100 μl, and the injections were administered once every three days for 3 consecutive treatments.

### Statistical analysis

The data are presented as the mean ± standard deviation. Statistical analysis was performed using Statistical Package Social Sciences (SPSS) version 17.0 (SPSS, Cary, NC, USA). A value of *p*<0.05 was considered significant, and *p*<0.01 was considered highly significant.

## Abbreviations

oHSV-2: Oncolytic herpes simplex virus type 2
DRG: Dorsal root ganglion
HCMV: Human cytomegalovirus
EBV: Epstein-Barr virus
HHV-8: Human Herpes virus 8
TEM: Transmission electron microscopy
MTT: 3-(4,5-dimethyl-2-thiazolyl)-2,5-diphenyl-2-H-tetrazolium bromide
